# Supplementary material for: Validating Use of Electronic Health Data to Identify Patients with Urinary Tract Infections in Outpatient Settings
Source: Antibiotics (Basel). 2020 Aug 25;9(9):536. doi: 10.3390/antibiotics9090536 (PMC7558992; doi:10.3390/antibiotics9090536)
Supplement: Supplementary file 1 [file antibiotics-09-00536-s001.pdf]

Supplyment material

Article

Validating Use of Electronic Health Data to Identify Patients with Urinary Tract Infections in Outpatient Settings

George Germanos [1], Patrick Light [2], Roger Zoorob [1], Jason Salemi [1], Fareed Khan [1], Michael Hansen [1,*], Kalpana Gupta [3], Barbara Trautner [4,5] and Larissa Grigoryan [1]

1. Department of Family and Community Medicine, Baylor College of Medicine, Houston, TX 77030, USA; george@germanos.md (G.G.); roger.zoorob@bcm.edu (R.Z.); jsalemi@usf.edu (J.S.); fareed.khan@bcm.edu (F.K.); grigorya@bcm.edu (L.G.)
2. Baylor College of Medicine, Houston, TX 77030, USA; patrick.light@bcm.edu
3. Section of Infectious Diseases, Department of Medicine, Boston Veterans Affairs Healthcare System and Boston University School of Medicine, Boston, MA 02118, USA; kalpana.gupta@va.gov
4. Houston VA Center for Innovations in Quality, Effectiveness and Safety (IQuESt), Michael E. DeBakey Veterans Affairs Medical Center, Houston, TX 77030, USA; trautner@bcm.edu
5. Section of Infectious Diseases, Departments of Medicine and Surgery, Baylor College of Medicine, Houston, TX 77030, USA; trautner@bcm.edu

* Correspondence: mahansen@bcm.edu; Tel.: +1 (713) 798-0114

Received: 21 July 2020; Accepted: 24 August 2020; Published: date

Table S1. ICD-10 [1] code(s) for each diagnosis and symptom.

Visit Classifications	ICD-10 [1] Codes
UTI Related Diagnoses	
Cystitis	N30
Acute Cystitis	N30.0, N30.00, N30.01
Other Chronic Cystitis	N30.2, N30.20, N30.21
Other Cystitis	N30.8, N30.80, N30.81
Cystitis Unspecified	N30.9, N30.90, N30.91
UTI (site not specified)	N39.0
Acute Pyelonephritis	N10
Nonobstructive Reflux-Associated Chronic Pyelonephritis	N11.0
Chronic Obstructive Pyelonephritis	N11.1
Pyonephritis	N13.6
UTI Related Symptoms	
Pain Associated with Micturition	R30
Dysuria	R30.0
Frequency of Micturition	R35.0
Urgency of Urination	R39.15

[1] ICD-10 = International Classification of Disease, tenth edition.

Table S2. Visit diagnosis codes.

	**Overall** ***n* = 829**	**Medicine [1]** ***n* = 681**	**Urology** ***n* = 148**	***p*-value [2]**
Acute cystitis	121 (11.3)	112 (12.1)	9 (5.6)	**0.024**
UTI, site not specified	694 (63.9)	562 (60.6)	132 (82.5)	**<0.001**
Acute pyelonephritis	6 (0.6)	4 (0.4)	2 (1.3)	0.48
Other[3]	8 (0.8)	3 (0.3)	5 (3.1)	**<0.006**

[1] Medicine includes the departments of Family Medicine and Internal Medicine. [2] p−values refer to chi-square tests. [3] Other includes other chronic cystitis, other cystitis, and cystitis unspecified. No diagnosis codes for nonobstructive reflux-associated chronic pyelonephritis, chronic obstructive pyelonephritis, or pyonephrosis were found.

Table S3. Visit symptom codes.

	**Overall** *n* **= 1233**	**Medicine [1]** *n* **= 1199**	**Urology** *n* **= 34**	***p*-value[2]**
Pain associated with micturition	524 (48.2)	515 (55.6)	9 (5.6)	**<0.001**
Dysuria	523 (48.1)	515 (55.6)	8 (5.0)	**<0.001**
Frequency of micturition	155 (14.3)	147 (15.9)	8 (5.0)	**<0.001**
Urgency of urination	31 (2.9)	22 (2.4)	9 (5.6)	**0.043**

[1] Medicine includes the departments of Family Medicine and Internal Medicine. [2] *p*-values refer to chi-square tests.

Table S4. Number of visits.

	**Frequency**	**Percent**	**Cumulative Frequency**	**Cumulative Percent**
1	800	86.67	800	86.01
2	97	10.51	897	97.18
3	20	2.17	917	99.35
4	1	0.11	918	99.46
5	3	0.33	921	99.79
7	2	0.22	923	100.00
